# Lessons Learned From Implementing Prospective, Multicountry Mixed-Methods Evaluations for Gavi and the Global Fund

**DOI:** 10.9745/GHSP-D-20-00126

**Published:** 2020-12-23

**Authors:** Emily Carnahan, Nikki Gurley, Gilbert Asiimwe, Baltazar Chilundo, Herbert C. Duber, Adama Faye, Carol Kamya, Godefroid Mpanya, Shakilah Nagasha, David Phillips, Nicole Salisbury, Jessica Shearer, Katharine Shelley, for the Gavi Full Country Evaluations Consortium; and Global Fund Prospective Country Evaluation Consortium

**Affiliations:** a PATH, Seattle, WA, USA.; b Infectious Diseases Research Collaboration, Kampala, Uganda.; c University of Eduardo Mondlane, Maputo, Mozambique.; d Institute for Health Metrics and Evaluation, University of Washington, Seattle, WA, USA.; e Department of Emergency Medicine, University of Washington, Seattle, WA, USA.; f Institut de Santé et Développement/University Cheikh Anta Diop, Dakar, Senegal.; g PATH, Kinshasa, Democratic Republic of the Congo.

## Abstract

**Introduction:**

As global health programs have become increasingly complex, corresponding evaluations must be designed to assess the full complexity of these programs. Gavi and the Global Fund have commissioned 2 such evaluations to assess the full spectrum of their investments using a prospective mixed-methods approach. We aim to describe lessons learned from implementing these evaluations.

**Methods:**

This article presents a synthesis of lessons learned based on the Gavi and Global Fund prospective mixed-methods evaluations, with each evaluation considered a case study. The lessons are based on the evaluation team’s experience from over 7 years (2013–2020) implementing these evaluations. The Centers for Disease Control and Prevention Framework for Evaluation in Public Health was used to ground the identification of lessons learned.

**Results:**

We identified 5 lessons learned that build on existing evaluation best practices and include a mix of practical and conceptual considerations. The lessons cover the importance of (1) including an inception phase to engage stakeholders and inform a relevant, useful evaluation design; (2) aligning on the degree to which the evaluation is embedded in the program implementation; (3) monitoring programmatic, organizational, or contextual changes and adapting the evaluation accordingly; (4) hiring evaluators with mixed-methods expertise and using tools and approaches that facilitate mixing methods; and (5) contextualizing recommendations and clearly communicating their underlying strength of evidence.

**Conclusion:**

Global health initiatives, particularly those leveraging complex interventions, should consider embedding evaluations to understand how and why the programs are working. These initiatives can learn from the lessons presented here to inform the design and implementation of such evaluations.

## Abstract (precis)

Lessons learned from implementing evaluations for Gavi, the Vaccine Alliance and the Global Fund for AIDS, Tuberculosis, and Malaria can help inform the design and implementation of ongoing or future evaluations of complex interventions. We share 5 lessons distilled from over 7 years of experience implementing evaluations in 7 countries.

## Teaser

**Key Findings:**

- We present 5 key lessons distilled from 7 years of experience implementing evaluations in 7 countries, which include the importance of:
  1. Including an inception phase to engage stakeholders and inform a relevant, useful evaluation design
  2. Aligning on the degree to which the evaluation is embedded in the program implementation
  3. Monitoring programmatic, organizational, or contextual changes and adapting the evaluation accordingly
  4. Hiring evaluators with mixed-methods expertise and using tools and approaches that facilitate mixing methods
  5. Contextualizing recommendations and clearly communicating their underlying strength of evidence

**Key Implications:**

- Global health initiatives, particularly those funding or implementing complex interventions, should consider embedding evaluations to understand how and why the programs are working to adapt as necessary and maximize impact.
- Evaluators of complex interventions should continue to share lessons learned related to balancing stakeholder priorities, aligning on “breadth” versus “depth” of the evaluation scope and ensuring use of the evaluation findings.

## INTRODUCTION

Complex interventions—those composed of several interacting components, sometimes with nonlinear causal pathways—are widely used to tackle complex global health challenges.^1^ ^,^ ^2^ As programs and interventions have become increasingly multidimensional, corresponding evaluations must be designed to assess the full complexity of these programs. Consequently, evaluations may need to consider not only programmatic outcomes, but also other outputs and outcomes across the system to understand how to improve programs to achieve impact. The goal of these evaluations is to understand not only *what* happened as a result of the program, but crucially *why* the change occurred. This need has resulted in an increased use of mixed methods, emergence of prospective approaches, and increased emphasis on process evaluation.^3^ ^–^ ^5^

Gavi, the Vaccine Alliance (Gavi) and the Global Fund to Fight AIDS, Tuberculosis, and Malaria (the Global Fund) are large multilateral organizations funding country governments and partners to implement necessarily complex interventions to improve public health. In 2018, the funding disbursement of both organizations totaled nearly $USD 4.5 billion,^6^ ^,^ ^7^ which is being used for packages of programs including vaccine purchasing, cold chain improvements, malaria prevention programs, HIV treatment programs, tuberculosis control programs, and general health systems support. Each organization has commissioned prospective mixed-methods evaluations to examine the implementation, outcomes, and impact of these complex interventions. We define a prospective evaluation as an approach for examining implementation processes and interventions forward in time, which has several advantages over retrospective evaluation, including deeper exploration of local context and implementation barriers and facilitators, ability to monitor phases of intervention implementation, and flexibility built into the design to incorporate emerging evaluation questions. Mixed-methods approaches are increasingly recognized as critical for health systems research in low- and middle-income country contexts,^8^ but definitions are numerous and varied. We draw from Ozawa and Pongpirul,^8^ who define these approaches as evaluations that “intentionally integrate or combine quantitative and qualitative data to maximize the strengths of each, to answer questions that are inadequately answered by one approach.”

Gavi’s Evaluation Advisory Committee, a sub-committee of the Gavi Board composed of independent evaluation advisors, commissioned the Gavi Full Country Evaluations (FCE) from 2013 to 2018. The FCE was funded by Gavi and managed by the Monitoring and Evaluation (M&E) team within the Gavi Secretariat. The FCE aimed to identify drivers of vaccine coverage and equity, with an emphasis on Gavi’s support of national immunization programs.^9^ The Global Fund Technical Evaluation Reference Group (TERG), an independent advisory group of the Global Fund, commissioned the Global Fund Prospective Country Evaluation (PCE) from 2017 to 2021 and provides oversight through the TERG Secretariat. Like the FCE, the PCE aims to generate evidence on how Global Fund processes and policies are enacted in real time in countries to achieve Global Fund objectives.^10^

PATH and the Institute for Health Metrics and Evaluation (IHME) at the University of Washington have served as the global evaluation partners (GEPs) leading a consortium of country evaluation partners (CEPs) for the FCE and PCE. These evaluations cover the full spectrum of Gavi/Global Fund support, including linkages between inputs, activities, outputs, outcomes, and impact. A variety of data sources and methods are used to triangulate evidence including resource tracking, process evaluation (document review, meeting observation, and key informant interviews), root cause analysis, social network analysis, secondary data analysis, geospatial analyses, value-for-money assessments, and impact modeling (complete methods available elsewhere^11^ ^–^ ^13^). The evaluations aimed to understand how Gavi/Global Fund policies and processes translate into country-level implementation to provide actionable, relevant insights to improve program implementation. Both evaluations were conducted in multiple countries to produce country-specific and cross-country synthesis findings to meet the needs of country and global stakeholders. The findings have successfully influenced Gavi/Global Fund policies and processes, and it has been suggested that these types of evaluations can be used for other global financing mechanisms or initiatives.^14^

Our approach has shifted over time to reflect learnings gained through implementing these evaluations since 2013.^11^ This article adds to the existing evaluation literature, and it expands on a complementary article from the Zambia FCE team’s perspective^15^ by taking a broader cross-country view of lessons learned from 2 prospective mixed-methods evaluations. We present lessons learned across the evaluation life cycle to inform the implementation of ongoing or future complex evaluations.

## METHODS

To generate lessons learned, we utilized our experience conducting prospective mixed-methods evaluations as part of the Gavi FCE and the Global Fund PCE, considering each evaluation as a case study. Insights came primarily from individuals who were involved in the implementation of the evaluation, both GEPs (PATH and IHME) that oversaw the evaluations and conducted cross-country synthesis, and CEPs that were primarily responsible for data collection, analysis, and reporting in their country. The CEPs included research organizations, academic institutions, and nonprofit organizations based in the focus countries for each evaluation (FCE: Bangladesh, Mozambique, Uganda, Zambia; PCE: Democratic Republic of the Congo, Guatemala, Senegal, Uganda).

Throughout the evaluations, GEPs and CEPs generated insights through periodic internal after-action reviews and systematic reflection sessions for adaptive management.^16^ The GEPs categorized insights according to the Framework for Evaluation in Public Health (Figure)^17^ and compared our experience-based insights with existing best practices within the framework to elucidate critical differences. This framework was chosen for its straightforward, comprehensive summary of the evaluation life cycle and its widespread use. Its key steps included engaging stakeholders; describing the program; focusing evaluation design; gathering credible evidence; justifying conclusions; and ensuring use and sharing lessons.

**FIGURE.**
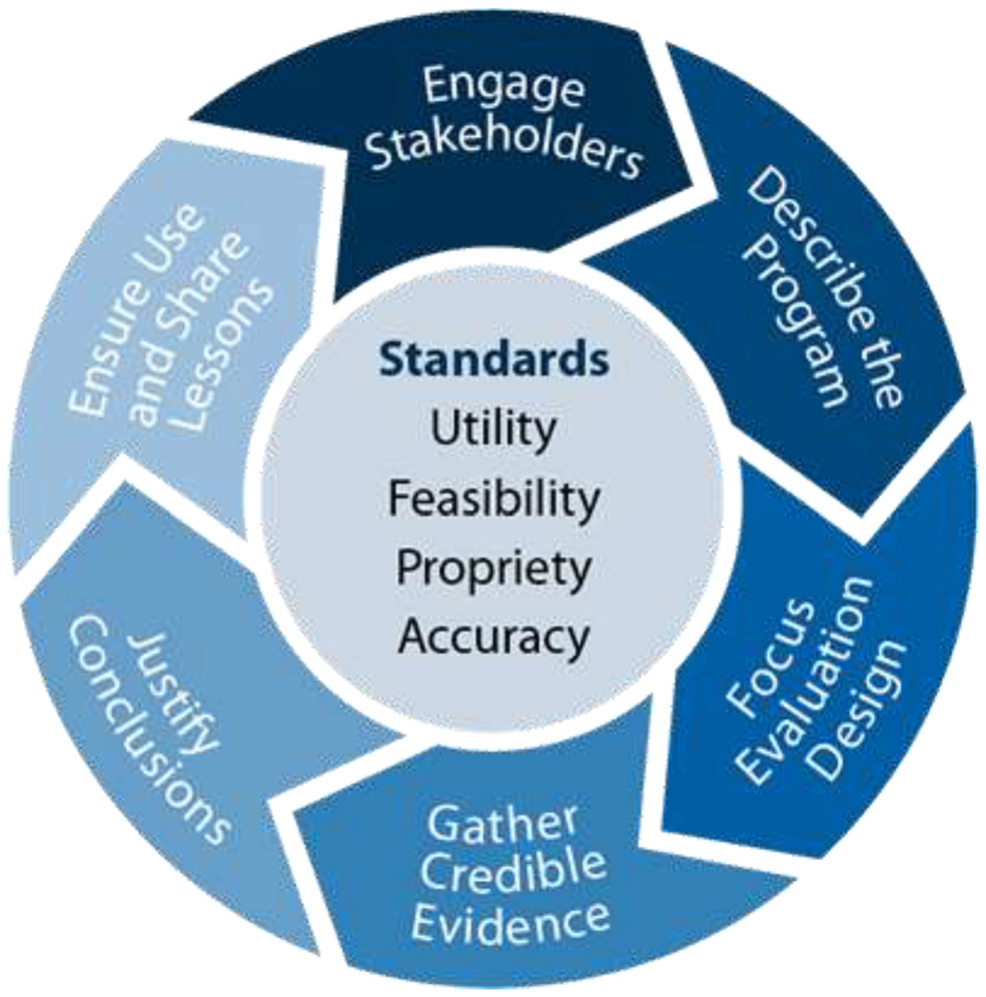
Centers for Disease Control and Prevention Framework for Evaluation in Public Health^17^

We report on the lessons learned that add new insights to existing best practices and are likely to be the most relevant to other teams undertaking complex prospective evaluations (Box). Other lessons learned that reinforce existing practices were omitted in the interest of space, although such omission does not mean they are not important in evaluation practice.

BOX Summary of Lessons Learned from Implementing Prospective, Mixed-Methods Evaluations

**Lesson 1**: For multistakeholder evaluations of complex interventions, the evaluation team and donors should include an inception phase to focus on stakeholder engagement and evaluation design. Support by high-ranking government officials and donor organizations during the inception phase can facilitate early stakeholder engagement.

**Lesson 2:** In a prospective process evaluation, the donor and evaluation team should align on the degree to which the evaluation is embedded in the program implementation; a quasi-embedded approach can balance objectivity and learning. Expectations for program stakeholders’ engagement in the evaluation should be clearly communicated by the evaluation team.

**Lesson 3:** In evaluations of complex interventions in which the programs, organizations, or contexts are constantly evolving, the evaluation team needs to continuously monitor changes and adapt the evaluation. The evaluation plan should be designed with enough flexibility to adjust evaluation questions and approaches to respond to changes; to support this, buy-in from the donor organization is essential.

**Lesson 4:** To successfully mix methods in a complex evaluation, evaluation teams should ideally include individuals with experience across methods or at minimum, co-locate individuals with various methods backgrounds. Tools and approaches—such as collaborative data review meetings, root cause analyses, and Tableau dashboards—can help to bridge any divide between quantitative and qualitative methods expertise.

**Lesson 5:** In evaluating complex adaptive interventions, the heightened need for attention to feasibility and context of recommendations means evaluators should clearly communicate the strength of evidence underlying each finding and should consider engaging stakeholders in the process of refining findings and recommendations.

## RESULTS

### Lesson 1: Include an Inception Phase to Engage Stakeholders

For multistakeholder evaluations of complex interventions, the evaluation team and donors should include an inception phase to focus on stakeholder engagement and evaluation design. Support by high-ranking government officials and donor organizations during the inception phase can facilitate early stakeholder engagement.

The FCE and PCE were each designed with an inception phase of 4 and 6 months, respectively. Given the complex nature of the evaluations, the inception phases were crucial to have dedicated time for a consultative and collaborative approach to engage stakeholders in developing a comprehensive understanding of the programs to be evaluated and refining the evaluation priorities and approach. Engaging stakeholders can improve evaluation design and relevance, facilitate data collection, and increase the likelihood that evaluation findings are used.^18^ ^–^ ^20^

Inception phases were crucial to have dedicated time for a consultative and collaborative approach to engage stakeholders in understanding the programs to be evaluated and refine the priorities and approach.

In the inception phases, we first relied on CEPs’ knowledge of the local context, reviews of relevant technical documents, and the organizational structure of key institutions to identify relevant stakeholders. We held face-to-face meetings with individuals or small groups of stakeholders to introduce the evaluation, which was essential to get buy-in from key government officials. These meetings were followed by half- or full-day kick-off meetings in each country with a wide range of stakeholders representing ministries of health and finance, implementing partners, technical partners, civil society, and Gavi/Global Fund. In many cases, the kick-off meetings were attended or endorsed by high-ranking stakeholders such as senior government officials who encouraged support for the evaluation. For example, the Permanent Secretaries of the Ministry of Health opened the FCE inception phase stakeholder meetings in Uganda and Zambia and the Minister of Health in Senegal presided over the opening ceremony of the PCE. Support from high-ranking officials paved the way for easier access to other government officials and partners for evaluation data collection and contributed to the sense of legitimacy of the evaluation, thereby improving the likelihood that findings would be used.

Support from funders also helped facilitate support for the evaluation. At the outset, a formal letter from the Gavi CEO was shared with country governments to endorse the FCE. During the inception phase, the Gavi M&E team and the evaluation team jointly met with key Ministry of Health personnel and other stakeholders, signaling Gavi’s support for the evaluation. The PCE did not have the same level of engagement from the Global Fund Secretariat in the inception phase, in part because the PCE was commissioned independently by the TERG. The limited early engagement from the Global Fund Secretariat resulted in early challenges for stakeholder buy-in, with downstream consequences in terms of accessing information, aligning the evaluation findings with decision making timelines at the Secretariat level, and ensuring widespread dissemination and use of synthesis findings.

### Lesson 2: Align on the Approach to Embedding the Evaluation in the Program Implementation

In a prospective process evaluation, the donor and evaluation team should align on the degree to which the evaluation is embedded in the program implementation; a quasi-embedded approach can balance objectivity and learning. Expectations for program stakeholders’ engagement in the evaluation should be clearly communicated by the evaluation team.

The continuum of potential evaluation designs ranges from a purely external evaluation that is entirely independent of the program implementation to a fully embedded evaluation that is internal to the implementation team.^21^ Purely external approaches may be more objective, but they have limited ability to understand changing program implementation, thereby potentially limiting the usefulness of the evaluation. A more embedded approach allows for more collaboration and feedback loops between the evaluation and program teams to adapt the evaluation to shifts in context, programs, or priorities.^21^ In evaluations of complex adaptive program implementation approaches, some degree of embeddedness to understand these shifts is appropriate. Process evaluation in particular requires collaborative and trusting relationships with stakeholders involved in the program implementation to facilitate access to information.^4^ And, as noted in lesson 1, engaging stakeholders in the evaluation can encourage uptake of evaluation findings.^18^ ^–^ ^20^ The FCE and PCE took a quasi-embedded approach to preserve evaluation objectivity while collaborating closely with stakeholders to support evaluation relevance, data access, and use of findings.

We used a quasi-embedded approach to preserve objectivity while collaborating with stakeholders to support evaluation relevance, data access, and use of findings.

The quasi-embedded approach can encourage timely learning through feedback loops between the evaluation teams and programs—and messaging the evaluation in this way, as a “learning platform,” helped increase stakeholder buy-in. During the initiation of the FCE and PCE, there were concerns that country stakeholders who were the most familiar with independent outcome evaluations would be resistant to the evaluation if they felt like they were being audited. Gavi anticipated this concern and emphasized to stakeholders that the FCE was not an evaluation of country programs per se, but of Gavi’s policies and processes, which helped to increase stakeholder buy-in. For the PCE, we shifted our framing to explain the evaluation as a learning platform that could provide support to stakeholders, help answer their priority evaluation questions, and provide evidence or recommendations to improve their program implementation. Many PCE stakeholders were initially unfamiliar with or had never engaged with a prospective evaluation in practice, so the concept of a learning platform was more intuitive. This framing also facilitated buy-in by differentiating the PCE from past Global Fund evaluations that some country stakeholders perceived as top-down audits rather than learning opportunities. The learning platform positioned CEPs and country stakeholders as partners in learning and opened the door for a collaborative relationship. This approach is in line with calls for more participatory and collaborative models for learning and evaluation in the international development field.^22^ ^–^ ^24^

This initial messaging of the FCE and PCE as partnerships focused on learning set the stage for a more collaborative relationship between evaluators and program implementers. The CEPs established close relationships with country stakeholders who were able to share documents and data, extend meeting invitations, and serve as key informants. Over time, the CEPs have become increasingly embedded in country programs, for example, being added to standing program meeting invites, which also meant they were on the email distribution list to receive meeting minutes and other key documents. While this involvement has enabled CEPs to track the unfolding processes in real time, gain access to essential documents and data sources, and share back emerging findings to improve program implementation, it has also made it challenging for CEPs to maintain evaluation independence (or a perception of independence). In some contexts, CEPs joining meetings solely as observers was not acceptable—they were expected to contribute if they wanted to keep being invited—thus, they shifted into participant observers.^25^ The Zambia FCE team highlighted their approach to provide meeting notes as a way of adding value,^15^ and across all CEPs, evaluator reflexivity was used to balance independence and embeddedness.^11^ ^,^ ^26^ ^–^ ^28^ Over time, the CEPs and stakeholders established shared expectations for engagement. This establishment of shared expectations—between evaluators and stakeholders, as well as evaluators and donors—should be discussed at the outset and revisited throughout the evaluation life cycle.

The initial messaging of the FCE and PCE led to a more collaborative relationship between evaluators and program implementers.

Although the FCE and PCE have used a quasi-embedded approach to balance objectivity and learning, there has been an ongoing tension in how to strike this balance in ensuring use of findings. Evidence uptake and knowledge translation rarely occur spontaneously and must be supported through a combination of “push,” “pull,” and “exchange” activities.^29^ As a result of the messaging of the evaluation as a learning platform and the embeddedness of evaluators, the evaluation team was perceived as being well positioned to engage stakeholders in these knowledge translation activities; however, encouraging the uptake of recommendations also risked compromising evaluation independence. To create accountability with stakeholders for acting on evaluation findings, while preserving evaluator independence, the Gavi Alliance provided an annual “management response” to the FCE findings and recommendations. The management response reported how Gavi had used each finding/recommendation.^30^ This approach could potentially be expanded to PCE country stakeholders or the Global Fund Secretariat to create more accountability for the use of findings, while maintaining independence of the evaluators. Ultimately, it is critical for the evaluation team and donor to align on the nature of collaboration and the role of the evaluation team at the outset of the evaluation because it has implications for how evaluators are received and how data are collected, as well as the adoption of findings.

### Lesson 3: Continuously Monitor Changes and Adapt the Evaluation

In evaluations of complex interventions in which the programs, organizations, or contexts are constantly evolving, the evaluation team needs to continuously monitor changes and adapt the evaluation. The evaluation plan should be designed with enough flexibility to adjust evaluation questions and approaches to respond to changes; to support this, buy-in from the donor organization is essential.

An evaluation plan should be designed with enough flexibility to adjust questions and approaches to respond to changes.

During the inception phases, the evaluation teams directly engaged the intended users of the evaluation to inform the evaluation focus, priorities, and evaluation questions (consistent with evaluation best practices^20^ ^,^ ^21^). The initial terms of reference for both the FCE and PCE provided overarching strategic evaluation questions, which the evaluation teams translated into a list of country-specific and cross-country evaluation questions responsive to the organizational context during the inception phase. However, as learning institutions, Gavi and the Global Fund frequently update processes and policies, which may affect evaluation context, objectives, and priorities throughout the course of the evaluation. (For example, Global Fund’s Operational Policy Manual^31^ undergoes numerous revisions throughout the year.) Thus, understanding the program and designing a responsive evaluation was not limited to the inception phase but required an ongoing assessment as to how the program was evolving, more consistent with developmental evaluation approaches.^32^ ^,^ ^33^

The quasi-embedded approach of the CEPs facilitated program monitoring at the country level, as did collaborative relationships with the Gavi M&E team and Global Fund TERG at the global level. Table 1 summarizes the approaches taken by the FCE/PCE teams to maintain direct access to stakeholders who could provide insights on the changes to Gavi/Global Fund policies and processes. Weekly calls with the Gavi M&E team and TERG Secretariat were helpful to regularly solicit updates related to policies, processes, or strategies, and buy-in from the donor organization is critical in supporting the evaluation team to fully engage with its staff (e.g., the Gavi Secretariat and Global Fund Secretariat).

**TABLE 1.** Approaches Taken by the Evaluation Teams to Engage With the Donor Organizations to Monitor Program Developments

**Gavi Full Country Evaluation**	**Global Fund Prospective Country Evaluation**
Weekly calls with the Gavi M&E team (GEP, CEP) KIIs with Secretariat staff throughout the year, with a concentration of KIIs during an annual in-person visit to Geneva (GEP) Semi-annual touchpoints with Gavi Senior Country Managers (GEP, CEP)	Weekly calls with the TERG Secretariat (GEP) Engagement with Secretariat staff at tri-annual TERG meetings (GEP, CEP) One-off phone calls with rotating Secretariat teams scheduled by the TERG Secretariat (GEP, CEP) Semi-annual touchpoints with Global Fund Country Teams (GEP, CEP)

Abbreviations: CEP, country evaluation partner; GEP, global evaluation partner; KII, key informant interview; M&E, monitoring and evaluation; TERG, technical evaluation reference group.

As context and priorities shifted throughout the course of the evaluation, the evaluation questions had to be updated to reflect these changes, identify emerging questions the evaluation could help to address, and ensure evaluation questions are useful. Buy-in from and engagement of the donor organization and other Secretariat staff was essential to ensure relevance of the updated evaluation questions, and the approaches summarized in Table 1 served as an opportunity to validate revised evaluation questions. This ongoing monitoring of the program context and discussion of priorities resulted in the adaptation and revision of evaluation questions, and ultimately a more flexible evaluation design. Two examples of how the PCE adapted evaluation questions based on shifts at the country level and global level are included in Table 2. Although it was necessary to design the evaluation to respond to the changing program context and priorities, we experienced pros and cons associated with designing the evaluation to encourage flexibility and adaptation over time (Table 3).

**TABLE 2.** Examples of Changing Prospective Country Evaluation Questions Due to Shifts at the Country and Global Levels

**Responsive to Country-Level Shift**	**Responsive to Global-Level Shift**
In Uganda, there was an unanticipated upsurge in malaria cases in 2019, so the Prospective Country Evaluation team added an evaluation question on whether and how Global Fund policies and structures enabled the country to respond.Findings indicated that several flexible aspects of the Global Fund business model, including modifications to procurement and supply chain plans, facilitated the country’s response to the malaria upsurge.	In 2020, the Grant Portfolio Solutions team at the Global Fund requested inputs about challenges related to Global Fund monitoring and reporting processes and opportunities for improvement.The Prospective Country Evaluation was able to quickly incorporate new evaluation questions into the evaluation scope and shared cross-country findings to inform the Secretariat’s revised reporting guidance.

**TABLE 3.** Pros and Cons of a Flexible Evaluation Design

**Pros**	**Cons**
Is responsive to changing stakeholder needs, thereby increasing stakeholder buy-in and the likelihood findings will be used. Has the ability to adjust to unanticipated implementation delays to refocus on the most timely, relevant evaluation questions.	Can take months to get stakeholder consensus on priorities. Requires carefully balancing those stakeholder inputs while remaining objective. May mean that evaluation teams are developing evaluation tools in parallel to prospectively tracking a process that has already started. This may undermine the planning required for intentional mixed methods approaches.

As we have strengthened relationships with stakeholders, and stakeholders have a better understanding of the scope of the evaluations, this process of adaptation has become more organic, with stakeholder inputs on evaluation questions shared more proactively and ad hoc. The GEPs/CEPs have also become more adept at identifying evaluation priorities through ongoing process tracking, including areas of cross-country synthesis that are most relevant in informing changes to Gavi/Global Fund policies or processes.

### Lesson 4: Include People With Mixed-Methods Expertise on the Evaluation Team

To successfully mix methods in a complex evaluation, evaluation teams should ideally include individuals with experience across methods, or at minimum co-locate individuals with various methods backgrounds. Tools and approaches, such as collaborative data review meetings, root cause analyses, and Tableau dashboards, can help to bridge any divide between quantitative and qualitative methods expertise.

To foster mixed-methods analysis, we learned that our teams (GEP and CEP) worked best when team members encompassed various disciplinary and methods backgrounds, were co-located, and used collaborative approaches to data interpretation and synthesis. Without conscious attention to team composition and processes, we found that mixing of methods and paradigms was difficult to achieve.

Without conscious attention to team composition and processes, we found that mixing of methods and paradigms was difficult to achieve.

Across the consortia, a range of staffing models were represented. Some CEPs had separate quantitative modeling and process evaluation teams, while others had integrated multidisciplinary teams. Aiming for a multidisciplinary team, preferably with multiple transdisciplinary staff that had “crossover” between methods expertise proved most successful. In cases in which CEP teams were divided methodologically, co-locating team members helped to ensure more regular full team meetings to review and triangulate emerging evidence, if not full mixing of methods.

To achieve true mixing of methods and paradigms, it is necessary to have both a well-integrated team with diverse expertise, as well as established procedures and processes for dialogue and analysis. While conducting a mixed-methods evaluation has been an ongoing challenge for some teams, the evaluations have adopted tools and approaches to help bridge the gap between quantitative and qualitative approaches. Using collaborative and interactive processes is valuable in facilitating mixed-methods analysis of the data and interpretation of findings. The PCE held joint CEP-GEP data review conference calls (approximately bimonthly) to share updated quantitative analyses, discuss data quality issues and resolutions, and identify opportunities for further triangulation with process evaluation evidence or the need for additional data collection. To further facilitate collective analysis, GEP and CEP held joint in-person analysis and report writing workshops 2 or 3 times per year, in addition to cross-country synthesis workshops at least once per year. The limitation in a more collaborative analysis process is the time and cost of engaging all evaluation partners—it is a dynamic and (potentially) nonlinear process that is best served by face-to-face interaction and may take substantive time.

In terms of tools, root cause analysis was a particularly effective analytic tool as it encouraged participants to incorporate all the available data—qualitative and quantitative—and iteratively explore hypotheses collaboratively. (Example FCE root cause analyses have been shared elsewhere.^11^ ^,^ ^15^) Similarly, Tableau dashboards were a useful tool to support interpretation of quantitative data among team members with a range of quantitative data skillsets; all team members had access to the dashboards and would look at the quantitative results to generate questions for qualitative follow-up. For example, the PCE visualized quantitative data from Global Fund grant revisions to understand budgetary shifts, and then generated key informant interview questions to understand why the shifts occurred and how they were affecting implementation activities.

Finally, we also learned that mixed-methods approaches can be more intentionally incorporated by starting from the evaluation question phrasing. Over time, we shifted to evaluation questions that encouraged mixed-methods data collection and analysis, such as “whether, why, and how does X outcome occur.” For example, the Uganda FCE team asked the question: “What is the effectiveness, efficiency, and country ownership of national immunization partnerships and their contribution to program performance?” This encouraged a mixed-methods approach that included social network mapping, document review, and qualitative interviews to understand the structure and added value of the partnership working on the Gavi HPV vaccine application.^34^

### Lesson 5: Contextualize Recommendations and Clearly Communicate Strength of Evidence

In evaluating complex adaptive interventions, the heightened need for attention to feasibility and context of recommendations means evaluators should clearly communicate the strength of evidence underlying each finding and should consider engaging stakeholders in the process of refining findings and recommendations.

Evaluators should clearly communicate the strength of evidence for each finding and consider engaging stakeholders in refining findings and recommendations.

Existing best practices focus on enhancing credibility of conclusions by ensuring data are analyzed and systematically interpreted, findings are directly linked to evidence and informed by stakeholder standards, and resulting recommendations are contextualized and actionable.^17^ While the evaluator’s role is to justify the evaluation conclusions, engaging stakeholders in the process presents a potential opportunity to further contextualize the findings and facilitate evidence use.^18^ The FCE/PCE teams shared preliminary findings with stakeholders for review to ensure we were reporting full and accurate information. Occasionally, these reviews would motivate stakeholders to share additional evidence to be incorporated. In determining when to share emerging findings, the evaluation team must balance the opportunity to gather additional insight from stakeholder reviews with the potential risk of sharing early findings with insufficient evidence that could undermine evaluators’ credibility.

Additionally, it is important to convey the strength of evidence underlying evaluation conclusions so stakeholders trust the findings and associated recommendations. This is particularly true in a mixed-methods evaluation in which each finding relies on multiple data sources with varying quality. Moreover, in some settings stakeholders perceived findings based solely on qualitative evidence to be less rigorous than quantitative evidence. To clearly signal the strength of evidence, we developed a rubric informed by GRADE and other evidence rating systems^35^ that rated the evidence along a 4-point scale.^15^ However, while the GRADE rubric considers study design and rates randomized trials highly, our scale was limited to the types of evidence used in the FCE/PCE, so randomized trials were not feasible or fit-for-purpose. Our rubric considered the extent of triangulation between data sources and the quality of the sources. Table 4 shows the strength of evidence rating used in the PCE, and Simuyemba et al.^15^ shared the rubric used in the FCE. Each finding was published with a rating to communicate our confidence in the conclusion, accounting for data quality and triangulation.

**TABLE 4.** Global Fund Prospective Country Evaluation Strength of Evidence Rating

**Rank**	**Rationale**
1	The finding is supported by multiple data sources (good triangulation) that are generally of strong quality.
2	The finding is supported by multiple data sources (moderate triangulation) of lesser quality, or the finding is supported by fewer data sources of higher quality.
3	The finding is supported by few data sources (limited triangulation) of lesser quality.
4	The finding is supported by very limited evidence (single source) or by incomplete or unreliable evidence. In the context of this prospective evaluation, findings with this ranking may be preliminary or emerging, with active and ongoing data collection to follow up.

During the FCE, the evaluation team independently generated recommendations that were shared with global and country stakeholders. The PCE has taken the same approach, but in some PCE countries we have used annual dissemination meetings as an opportunity to iteratively refine the recommendations with stakeholders. This approach has been well received and may prove a promising practice to generate buy-in for acting on the recommendations.

## DISCUSSION

This article presents 5 lessons distilled from over 7 years of experience (2013–2020) implementing prospective mixed-methods evaluations of Gavi and the Global Fund in 7 countries. While country settings were highly variable, our experiences had some consistency, resulting in a mix of operational and practical “how to” considerations, alongside broader considerations that are sometimes more “art than science.”

The Framework for Evaluation in Public Health was a useful tool to ground the identification of lessons learned. However, while the framework suggests a distinct, linear process for evaluation, feedback loops existed between steps in practice, and some steps (e.g., stakeholder engagement) were a focus throughout the duration of the evaluations. Our lessons spanned steps in the evaluation life cycle—and are often interrelated and mutually reinforcing—and therefore we decided against presenting lessons learned aligned to specific steps in the Framework, instead emphasizing their cross-cutting nature.

Stakeholder engagement is a key theme that weaves many lessons together. In the FCE/PCE, the inception phase was the initial touchpoint to engage stakeholders (lesson 1), but strengthening relationships between evaluators and other stakeholders was an ongoing effort. The quasi-embedded approach (lesson 2) facilitated these relationships, particularly at the country level. And strong relationships—based on shared trust, collaboration, and learning—between the evaluators and stakeholders enabled program monitoring and evaluation adaptation (lesson 3), facilitated data access to inform a mixed-methods approach (lesson 4), and led to contextualized findings and recommendations (lesson 5).

Stakeholder engagement is a key theme that weaves many lessons together.

A second cross-cutting theme is the balance of objectivity and learning. In recent years, the evaluation discipline has come to embrace its role in adaptation and learning, and this has extended greater latitude for how evaluator reflexivity can allow independence coupled with learning.^26^ ^,^ ^36^ ^,^ ^37^ A spectrum of evaluation models are available, depending on the nature of interactions between program implementers and evaluators and the degree of embeddedness desired.^21^ In the FCE/PCE, the quasi-embedded evaluation approach (lesson 2) allowed for timely monitoring of the program context to understand and respond to changing program needs (lesson 3). This quasi-embeddedness also allowed evaluators to communicate the strength of findings to inform stakeholders’ action (lesson 5). Ultimately, stakeholders should consider the level of objectivity and collaboration that would make an evaluation fit-for-purpose, and let that inform the appropriate degree of embeddedness in the evaluation design; there is no one-size-fits-all model for evaluation of complex interventions.

Another key theme across many of the lessons relates to the design and focus of the evaluations. Complex interventions and evaluations of those interventions often include multiple stakeholder audiences with different evaluation priorities or goals. The inception phase (lesson 1) should help define the scope of the evaluation and bring clarity to stakeholders on what the evaluation will—and, importantly, will not—address. However, we also advocate for flexibility in the evaluation design (lesson 3) to adjust evaluation questions based on shifting context, priorities, or implementation approaches. A flexible evaluation design has pros and cons (as noted in Table 3), and this is an area of continued learning for the PCE, as is discussed further in the Implications section. A flexible evaluation design requires an ongoing process of aligning and realigning on the evaluation questions and scope across multiple stakeholder audiences. Overall, it has been important to continuously engage with stakeholders so they know which questions have been prioritized and what types of findings to anticipate.

Continuously engaging with stakeholders has been important so they know which questions have been prioritized and what types of findings to anticipate.

### Implications and Future Research

Our evaluation approach has shifted over more than 7 years of implementation.^11^ As we have refined our approach, areas still remain in which we continue to learn and further refinement is required. These include balancing stakeholder priorities, aligning on “breadth” versus “depth” of the evaluation scope, and identifying approaches to ensure use of the evaluation findings.

In terms of balancing stakeholder priorities, these multilevel, multistakeholder evaluations were designed to meet the needs of a range of country and global stakeholders. It has proven challenging to design an evaluation that balances the diverse needs of distinct groups of primary users with differential interests and power. CEPs have been more likely to prioritize evaluation questions identified by country stakeholders to be responsive to country needs. Conversely, our oversight points of contact at Gavi and Global Fund have been more likely to prioritize cross-country evaluation questions that can directly inform policies or strategies or are responsive to their funders and board members. With limited resources, if tradeoffs needed to be made between being responsive to global versus country priorities, it was not clear which to prioritize. Striking a balance between stakeholder priorities has been an ongoing challenge.

A second area of continued learning is how to align stakeholders on the tradeoffs between covering a wide breadth of topics versus going in depth on fewer topics. In setting the evaluation questions, the FCE and PCE teams have continuously navigated the tradeoffs between depth versus breadth of the evaluation scope. Process tracking (through document review, meeting observation, and key informant interviews) was intended to understand the breadth of activities, and based on stakeholder priorities and emerging findings, evaluation questions could be identified for further in-depth analysis. However, in practice, it has been challenging for CEPs to track all the processes unfolding—particularly for the PCE since it covers 3 large disease programs, with many stakeholders and grant activities. Over time, both the FCE and PCE shifted toward less breadth and more depth, with more focused evaluation questions and analytical approaches. On reflection, it was important for the evaluation teams to start with a broad scope to understand all the interrelated components of the complex interventions; with this understanding in place, it was possible to narrow the evaluation focus to go further in depth without losing the wider context.

Finally, we continue to test and refine our approach to ensuring use of the evaluation findings among target audiences. Lessons 1 and 2 highlight our approach to engaging with stakeholders, which engenders buy-in to the evaluation and uptake of findings. Best practices emphasize tailoring dissemination strategies to stakeholders and providing knowledge translation support^18^ ^,^ ^38^; however, the FCE and PCE teams have had limited resources and capacity to support this effort. Our more formalized dissemination approaches have focused primarily on annual written reports and annual country-based dissemination meetings. Annual dissemination meetings have worked well to bring together a diverse set of stakeholders to discuss evaluation findings and recommendations and provide input on future evaluation priorities. However, the timing of annual meetings and reports may not align with program implementation timelines or decision-making windows. Thus, it is important to have multiple modes of disseminating findings. We recommend that future evaluations are resourced to support knowledge translation and more timely sharing of emerging findings (e.g., through shorter policy briefs, evaluation team engagement in program meetings) to fully take advantage of the learning platform.

We recommend that future evaluations are resourced to support knowledge translation and more timely sharing of emerging findings.

### Limitations

The content for this article draws solely from the experiences of the FCE and PCE evaluation teams, meaning the lessons do not directly incorporate the perspectives of other key stakeholders (e.g., Gavi, Global Fund, Ministries of Health) on what aspects of the evaluation worked well and added value versus those needing further refinement. Furthermore, the lessons presented are not exhaustive; the authors’ judgment was used to determine which lessons were most novel and important to highlight. Another potential limitation is that lessons are drawn only from the FCE and PCE cases, which are unique evaluations in scale and scope and not necessarily generalizable. However, the case uniqueness also suggests lessons may be particularly relevant to other large global health initiatives with interest in establishing similar multiyear, independent prospective evaluations of their investments, policies, and processes.

## CONCLUSION

A key benefit of prospective mixed-methods evaluations is the opportunity for dynamic and continuous learning because data are collected while implementation unfolds. This means that evaluators can identify what is working or not working and explore why. Although this type of evaluation has added value to Gavi’s and Global Fund’s understanding of their programs, this approach is a new way of working for many evaluators, donors, and other stakeholders, meaning it can take time to understand and engage with. Therefore, this article presents 5 lessons distilled from over 7 years of experience (2013–2020) implementing prospective, mixed-methods evaluations of Gavi and the Global Fund in 7 countries. Our aim in writing this article was to reflect on and share key lessons that we hope can inform the design and implementation of future prospective evaluations of large-scale, complex global health initiatives. Such global health initiatives, particularly those leveraging complex interventions, should consider embedding evaluations to understand how and why the programs are working to adapt as necessary and maximize impact.

## Notes

**Peer Reviewed**

**First published online:** November 11, 2020
